# Characterization of a *fosA3* Carrying IncC–IncN Plasmid From a Multidrug-Resistant ST17 *Salmonella* Indiana Isolate

**DOI:** 10.3389/fmicb.2020.01582

**Published:** 2020-07-21

**Authors:** Li-Juan Zhang, Xi-Xi Gu, Jie Zhang, Ling Yang, Yue-Wei Lu, Liang-Xing Fang, Hong-Xia Jiang

**Affiliations:** ^1^Guangdong Provincial Key Laboratory of Veterinary Pharmaceutics Development and Safety Evaluation, College of Veterinary Medicine, South China Agricultural University, Guangzhou, China; ^2^Guangdong Laboratory for Lingnan Modern Agriculture, Guangzhou, China

**Keywords:** *Salmonella*, IncC–IncN plasmid, *fosA3*, *bla*_CTX–M–14_, multidrug-resistant

## Abstract

The aim of this study was to investigate the characteristics of a *fosA3* carrying IncC–IncN plasmid from a multidrug-resistant *Salmonella* isolate HNK130. HNK130 was isolated from a chicken and identified as ST17 *Salmonella enterica* serovar Indiana and exhibited resistance to 13 antibiotics including the cephalosporins and fosfomycin. S1 nuclease pulsed-field gel electrophoresis and Southern blot assays revealed that HNK130 harbored only one ∼180-kb plasmid carrying *fosA3* and *bla*_CTX–M–14_, which was not transferable via conjugation. We further examined 107 *Escherichia coli* electro-transformants and identified 3 different plasmid variants, pT-HNK130-1 (69), pT-HNK130-2 (15), and pT-HNK130-3 (23), in which pT-HNK130-1 seemed to be the same as the plasmid harbored in HNK130. We completely sequenced an example of each of these variants, and all three variants were IncC–IncN multi-incompatible plasmid and showed a mosaic structure. The *fosA3* gene was present in all three and bounded by IS*26* elements in the same orientation (IS*26*-322bp-*fosA3*-1758bp-IS*26*) that could form a minicircle containing *fosA3*. The *bla*_CTX–M–14_ gene was located within an IS*15DI-*ΔIS*15DI-iroN-*IS*903B-bla*_CTX–M–14_*-*ΔIS*Ecp1-*IS*26* structure separated from the *fosA3* gene in pT-HNK130-1, but was adjacent to *fosA3* in pT-HNK130-3 in an inverted orientation. Linear comparison of the three variants showed that pT-HNK130-2 and pT-HNK130-3 resulted from the sequence deletion and inversion of pT-HNK130-1. Stability tests demonstrated that pT-HNK130-1 and pT-HNK130-3 could be stably maintained in the transformants without antibiotic selection but pT-HNK130-2 was unstable. This is the first description of an IncC–IncN hybrid plasmid from an ST17 *S.* Indiana strain and indicates that this plasmid may further facilitate dissemination of fosfomycin and cephalosporin resistance in *Salmonella.*

## Introduction

Fosfomycin is a natural broad-spectrum antibiotic with activity against both Gram-positive and Gram-negative bacteria and has a unique mechanism of action and can synergize with other antibiotics including β-lactams, aminoglycosides, and fluoroquinolones (Raz, 2015; He et al., 2017). The recent rapid increase of multidrug-resistant (MDR) bacteria has limited antibiotic treatment options for infections caused by these pathogens and fosfomycin has become a new choice for the treatment of MDR bacterial infections (Raz, 2015).

Although the use of fosfomycin is prohibited in animals in China, a plasmid-mediated fosfomycin resistance gene *fosA3* is often observed in *Escherichia coli* isolates from pets and food animals (Hou et al., 2012, 2013; He et al., 2013, 2017). Furthermore, *fosA3* has been found in association with *bla*_CTX–M_, *rmtB*, *floR*, *bla*_NDM_, and other antibiotic resistance genes (ARGs) on disseminated plasmids, and this has promoted further spread of *fosA3* (Hou et al., 2012, 2013; He et al., 2013, 2017; Villa et al., 2015; Hadziabdic et al., 2018). At present, *fosA3* has been detected primarily in *E. coli* isolates from pets, pigs, wild animals, and food animals at rates of 1.1 to 9.0% (Hou et al., 2012, 2013; Ho et al., 2013). However, there are only a few reports that have identified *fosA3* in *Salmonella* isolates (Lin and Chen, 2015; Villa et al., 2015; Wang et al., 2017; Hadziabdic et al., 2018; Fang et al., 2019).

*Salmonella* is an important global food-borne pathogen of both humans and livestock and 70–80% of annual foodborne diseases in China are attributed to *Salmonella* infections (Coburn et al., 2007; Wang and Zhang, 2013). Recent reports of *Salmonella* isolates have found *fosA3* in association with *bla*_NDM–1_ and *bla*_CMY–16_ on an IncA/C plasmid (Villa et al., 2015; Hadziabdic et al., 2018) as well as with *mcr-1*, *oqxAB*, and *rmtB* on HI2 and F33:A-:B- plasmids (Wong et al., 2016; Wang et al., 2017; Fang et al., 2019). However, there is still little information about the prevalence and characteristics of the *fosA3* gene among *Salmonella* isolates. Herein, we report the emergence of an IncC–IncN [IncC, previously termed IncA/C_2_ (Harmer and Hall, 2016)] plasmid that possesses *fosA3*, *bla*_CTX–M–14_, the heavy metal resistance genes *sil* and *mer* as well as other ARGs in *Salmonella* isolates from chickens in China. This is the first description of an IncC–IncN hybrid plasmid from an ST17 *Salmonella* Indiana strain, and this hybrid plasmid may have originated from a variety of different plasmids. Our findings also provide important insights into plasmid evolution and the dissemination of fosfomycin resistance in *Salmonella*.

## Materials and Methods

### Strains

*Salmonella* strain HNK130 was isolated from fecal swabs collected from healthy chickens in Henan province of China during 2014 as reported in the previous study (Zhang et al., 2016). In our previous research on the monitoring of drug resistance of *Salmonella* from food animals (Zhang et al., 2016), this strain was found to be highly resistant to fosfomycin (MIC > 512 μg/ml). The presence of *fosA3* and other resistance genes of this strain was screened by PCR amplification using primers described previously (Hou et al., 2012; Li et al., 2015; Lin and Chen, 2015). This strain was then serotyped using slide agglutination with hyperimmune sera (S & A Company, Bangkok, Thailand) and the results were interpreted according to the Kauffman–White scheme as described previously (Zhang et al., 2016). Multilocus sequence typing (MLST) of this strain was performed by PCR and DNA sequence analysis of seven housekeeping genes *aroC*, *dnaN*, *hemD*, *hisD*, *purE*, *sucA*, and *thrA* to determine the allelic profiles using software available online.^1^

To detect the prevalence of *fosA3*-carrying IncC–IncN hybrid plasmid among *Salmonella* isolates, a total of 288 *Salmonella* isolates collected and preserved in our laboratory during 2009–2014 were used for screening. Out of the 288 isolates, 126 (44%) isolates were recovered from a total of 1728 pork samples collected from a large-scale slaughterhouse in Guangdong, China, during the period of April 2013 to April 2014. Among the remaining 162 (56%) isolates, except for 3 (1.0%) that were obtained from 180 chicken fecal samples collected from Guangdong, China in October 2009, the other 159 (55%) strains were all isolated from 3850 non-repetitive fecal swabs collected from healthy chickens and pigs in Guangdong, Shandong, Henan, and Hubei provinces of China during 2014. Of these samples collected in 2014, 2090 samples were from chickens and 1760 samples were from pigs; the *Salmonella* isolates obtained from chickens and pigs were 90 and 69, respectively.

### Antimicrobial Susceptibility Testing

The minimum inhibitory concentrations (MICs) of this strain and its transformants for 15 different antibiotics including ampicillin (AMP), cefotaxime (CTX), cefoxitin (CXT), ceftiofur (CTF), ceftriaxone (CTR), nalidixic acid (NAL), ciprofloxacin (CIP), enrofloxacin (ENR), kanamycin (KAN), gentamycin (GEN), amikacin (AMK), tetracycline (TET), chloramphenicol (CHL), florfenicol (FLF), and fosfomycin (FOM) were determined by the agar dilution method according to the CLSI guidelines (CLSI, 2018). *E. coli* ATCC25922 served as a quality control strain. MIC of AgNO_3_ was determined by broth microdilution method in an aerobic atmosphere as previously described (Fang et al., 2016); *E. coli* C600 and *E. coli* DH5α was used as a reference strain.

### Conjugation and Transformation Assays

To test the transferability of the *fosA3*-positive plasmid, both conjugation experiments and transformation experiments were performed. Conjugation experiments were conducted for strain HNK130 using the sodium azide–resistant *E. coli* J53 as the recipient by both liquid and solid mating-out assay in Luria-Bertani medium (LB-medium). Transconjugants were selected on MacConkey agar supplemented with 300 mg/L sodium azide and 200 mg/L fosfomycin. For transformation experiments, plasmid DNA from the *fosA3*-positive strains were extracted using Qiagen Plasmid Midi Kits according to the manufacturer’s instruction (Qiagen, Hilden, Germany). Purified plasmids were transformed into *E. coli* DH5α (Takara, Dalian, China). Selection of transformants was performed on MacConkey agar containing 200 mg/L fosfomycin, 1 mg/L cefotaxime, and both 200 mg/L fosfomycin and 1 mg/L cefotaxime. The presence of the *fosA3* and *bla*_CTX–M–9G_ genes in the transconjugants and transformants were confirmed by PCR.

### Plasmid Characterization

PCR-based replicon typing (PBRT) was performed on transformants using primers as described previously (Carattoli et al., 2005). To determine the location of *fosA3* and *bla*_CTX–M–14_, pulsed-field gel electrophoresis (PFGE) with S1 nuclease (Takara) digestion of whole genomic DNA was carried out as described previously (Barton et al., 1995). The resulting gels were analyzed by Southern transfer and probing with a DIG-labeled *fosA3* gene and *bla*_CTX–M–9G_ gene fragment according to the manufacturer’s instructions using a DIG High Prime DNA Labeling and Detection Starter Kit I (Roche, Mannheim, Germany).

### Plasmid Sequencing and Bioinformatics Analyses

The complete sequence of plasmid was determined by sequencing using long-read MinION (Oxford Nanopore) and short-read Illumina MiSeq technologies. Plasmid DNA was extracted from transformants with the Qiagen Plasmid Midi Kit (Qiagen, Courtaboeuf, France) according to the manufacturer’s instructions and then sent to Biomarker Technologies for sequencing (Biomarker, Beijing, China). The long reads were generated by Oxford Nanopore MinION flowcell R9.4 (Li et al., 2018) with a depth more than 300 times and the 150 bp paired-end short reads were generated by Illumina MiSeq system (Illumina, San Diego, CA, United States) with a depth more than 100 times. The MinION reads were filtered using Filtlong (version 0.2.0) to remove any reads <2000 bp, followed by removal of the lowest 10% of reads by quality. Complete sequence assembly was performed with Canu version 1.5 (Koren et al., 2017) using a combination of short and long reads, followed by error correction by Pilon version 1.12 (Walker et al., 2014). Gene prediction and annotation were done with RAST,^2^ and IS finder.^3^ Plasmid replicon types and the plasmid MLST (pMLST) were analyzed using the CGE server.^4^ Sequences were analyzed and compared using BLAST,^5^ and map generation was performed using Easyfig (version 2.3) (Sullivan et al., 2011) and BRIG (Alikhan et al., 2011).

### Plasmid Stability Testing

The stability of HNK130 and 3 *fosA3*-positive transformants (T-HNK130-1,-2,-3) was studied by passage in antibiotic-free Luria broth (LB) as previously described (Sandegren et al., 2012). Three separate cultures of each strain carrying the plasmid were grown in 3 ml antibiotic-free LB overnight at 37°C, followed by serial passage of 3 μl overnight culture into 3 ml LB each day, yielding 10 generations for each strain, lasting 20 days. Every 2 days, the cultures were collected and serially diluted using 0.85% saline and grown on LB agar in the absence of antibiotics. Then, 50 colonies were screened on LB agar plates with or without fosfomycin to determine the fraction of plasmid-containing cells, and the plasmid loss was verified by PCR amplification of *fosA3* and *bla*_CTX–M–9G_.

### Accession Number(s)

The plasmid sequences of pT-HNK130-1, pT-HNK130-2, and pT-HNK130-3 have been deposited in GenBank with accession numbers CP045742, CP046032, and CP047128, respectively. We also have submitted the raw reads (both Illumina and Nanorope) of pT-HNK130-1, pT-HNK130-2, and pT-HNK130-3 to the Sequence Read Archive (SRA); the SRA accession was PRJNA631125.

## Results

### Characterization of *fosA3*-Bearing *Salmonella* HNK130

**FIGURE 1:**
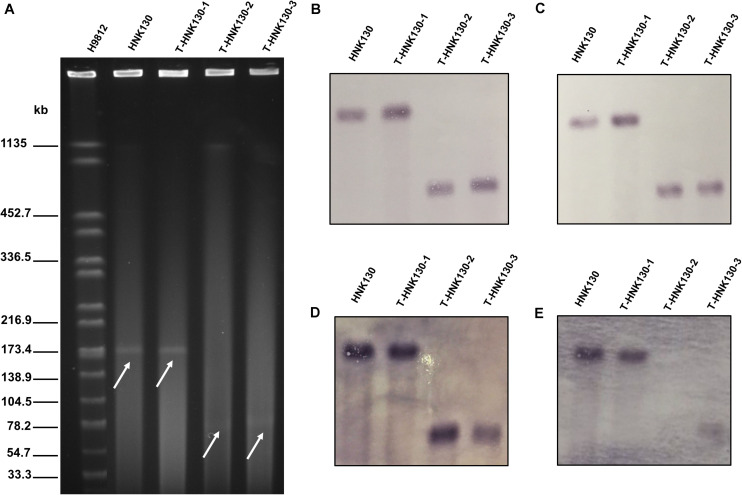
Analysis of HNK130 and its *fosA*_3_-positive transformants. **(A)** S1-PFGE; **(B)** Southern hybridization with IncC-specific probe; **(C)** Southern hybridization with IncN-specific probe; **(D)** Southern hybridization with *fosA3*-specific probe; **(E)** Southern hybridization with *bla*_CTX–9G_-specific probe.

The *Salmonella* strain HNK130 was found to be ST17 *S. enterica* serovar Indiana and exhibited an MDR profile for a wide range of antimicrobial agents, including the cephalosporins, aminoglycosides, tetracyclines, and fluoroquinolones (Table 1). The metal susceptibility testing showed that HNK130 and its transformants had the MIC of AgNO_3_ higher than that for the recipient *E. coli* C600 and *E. coli* DH5α (MIC_AgNO3_ = 0.015–0.03 mM vs. 0.008 mM) when compared with *E. coli* C600 and *E. coli* DH5α (Table 1). Screening for other resistance genes confirmed that this strain co-harbored *bla*_CTX–M–14_ and *floR*. S1-PFGE and hybridization revealed only one plasmid (∼180 kb) in the strain HNK130 and *fosA3* and *bla*_CTX–M–14_ were present on this plasmid (Figure 1).

**TABLE 1:** Characteristics of HNK130 and its transformants selected for this study.

**Strain**	**Resistance genes**	***fosA3* genetic environment**	**Plasmid size (kb)**	**Replicon type**	**MIC of AgNO_3_ (mM)**	**Antibiotic resistance patterns**
HNK130	*bla*_CTX–M–14_, *fosA*_3_, *floR*	B	∼180	C, N	0.03	AMP/CTX/CTF/CTR/CHL/FLF/KAN/STR/CIP/ENR/NAL/TET/FOM
THNK130-1	*bla*_CTX–M–14_, *fosA*_3_, *floR*	B	∼180	C, N	0.03	AMP/CTX/CTR/CHL/FLF/KAN/STR/NAL/TET/FOM
THNK130-2	*fosA*_3_, *floR*	A	∼80	C, N	0.015	CHL/FLF/KAN/STR/NAL/TET/FOM
THNK130-3	*bla*_CTX–M–14_, *fosA*_3_, *floR*	C	∼80	C, N	0.015	AMP/CTX/CTR/CHL/FLF/KAN/STR/NAL/TET/FOM

*AMP, ampicillin; CTX, cefotaxime; CTF, ceftiofur; CTR, ceftriaxone; CHL, chloramphenicol; FFF, florfenicol; KAN, kanamycin; STR, streptomycin; CIP, ciprofloxacin; ENR, enrofloxacin; NAL, nalidixic acid; TET, tetracycline; FOM, fosfomycin; MIC, minimum inhibitory concentration. A: Genetic environments of *fosA3* are shown in Supplementary Figure S3. T: transformant.*

Although multiple attempts to transfer the *fosA3*-bearing plasmid from the donor bacteria to *E. coli* J53 by conjugation were not successful, the fosfomycin resistance trait could be successfully transferred into *E. coli* DH5α via electro-transformation. We selected three to five clones for *bla*_CTX–M–9G_ and *fosA3* amplification by PCR to verify the transformants. Interestingly, after repeated verifications, we found two different types of transformants: group 1 contained both *fosA3* and *bla*_CTX–M–14_ while group 2 contained *fosA3* and lacked *bla*_CTX–M–14_. To further study the presence of two different types of transformants, we randomly selected 107 transformants for PCR detection. Among the 107 transformants, 86% (92/107) of them belong to group 1, and the remaining 14% (15/107) belong to group 2. Plasmid replicon typing revealed that all 107 transformants contained both IncC and IncN replicons.

We then randomly selected nine transformants from groups 1 and 3 from group 2 for S1-PFGE analysis. The nine transformants from group 1 possessed plasmids of two different sizes, i.e., 7 were ∼180 kb and the remaining 2 were ∼80 kb. The group 2 transformants possessed only ∼80 kb plasmids. We examined the genetic features of three of these plasmid variants and designated them as pT-HNK130-1 to pT-HNK130-3 from group 1 *fosA3*/*bla*_CTX–M–14_ positive ∼180 and ∼80 kb plasmids and from group 2 the *fosA3* positive ∼80 kb plasmid, respectively. We performed Southern hybridization with DIG-labeled *bla*_CTX–M–9G_, *fosA3*, IncC, and IncN-specific probe in transformants harbored pT-HNK130-1, pT-HNK130-2, and pT-HNK130-3 and showed that the plasmid replicon gene and the antibiotic resistance gene were co-located on the same plasmid in the all three transformants (Figure 1). In addition, based on S1-PFGE and plasmid replicon typing, the plasmid variant pT-HNK130-1 was the same as the plasmid in the wild-type strain.

We also assessed the stability of the three transformants and parent strain HNK130 in the absence of antibiotic selection. The *bla*_CTX–M–14_ and *fosA3* genes co-transferred plasmids (pT-HNK130-1 and pT-HNK130-3) were stably maintained in the transformants and plasmid loss did not occur. In contrast, the pT-HNK130-2 that contained only the *fosA3* gene was unstable and plasmid loss was associated with passage in the absence of antibiotic pressure (Supplementary Figure S1).

### Sequence Analysis of pT-HNK130-1, pT-HNK130-2, and pT-HNK130-3

**FIGURE 2:**
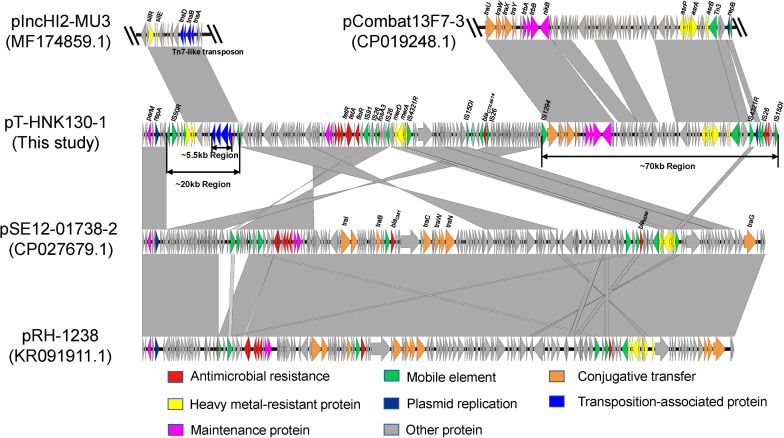
Sequence linear comparison between pT-HNK130-1, pCombat13F7-3 (GenBank accession no. CP019248.1), pIncHI2-MU3 (accession no. MF174859.1), pSE12-01738-2 (accession no. CP027679.1), and pRH-1238 (accession no. KR091911). Gray shading indicates shared regions with a high degree of homology. Genes are represented by arrows and are colored depending on gene function as depicted. Genes are color coded as follows: dark blue, replication; green, mobile element; red, antimicrobial resistance; yellow, heavy metal–resistant protein; blue, transposition-associated protein; orange, conjugative transfer; rose red, maintenance protein; gray, other protein.

Complete sequences of pT-HNK130-1, pT-HNK130-2, and pT-HNK130-3 were obtained to investigate the genetic features of the three variants and the genetic context of the *fosA3* gene and *bla*_CTX–M–14_ gene. The largest variant plasmid pT-HNK130-1 was 180,784 bp with a GC content of 51% and carried 255 open reading frames (ORFs). This plasmid belonged to the IncC and IncN incompatibility types. pT-HNK130-1 was 99.88% identical at 53% coverage to plasmid pSE12-01738-2 (CP027679.1), a *bla*_NDM–1_-carrying IncC plasmid derived from a *Salmonella* strain isolated in 2012 from a wild bird in Germany. pT-HNK130-1 also showed high similarity to pRH-1238 (KR091911.1, recovered from a *Salmonella* isolated from a migratory wild bird in Germany) with 53% coverage and 99.92% identity. pRH-1238 is the first completely sequenced *bla*_NDM–1_-*fosA3* IncC plasmid and pSE12-01738-2 is a derivative of pRH-1238. Sequence comparisons between pSE12-01738-2 and pT-HNK130-1 showed that in addition to missing and inverted portions of the part of IncC plasmid backbone, there were two regions of (∼70 and ∼20 kb, respectively) of pT-HNK130-1 that did not align to pSE12-01738-2 (Figure 2). The ∼70 kb region of pT-HNK130-1 included backbone elements that encoded functions for horizontal transfer and IncN plasmid replication. BLASTN results for further analysis of this region showed that it was 99.91% identical to the plasmid pCombat13F7-3(CP019248.1) at 77% coverage. pCombat13F7-3 was recovered from a clinical *E. coli* strain in Hong Kong and belonging to incompatibility groups N and I1. The ∼20 kb region of pT-HNK130-1 contained a Tn7-like transposon (∼5.55 kb) encompassing the *tns*ABCD genes and silver resistance genes *silR*, *silS*, and *silE* were related to pIncHI2-MU3(MF174859.1) with 86% coverage and 99.64% identity. Sequence analysis indicated that pT-HNK130-1 showed a mosaic structure and may have formed from a variety of plasmids (Figure 2).

**FIGURE 3:**
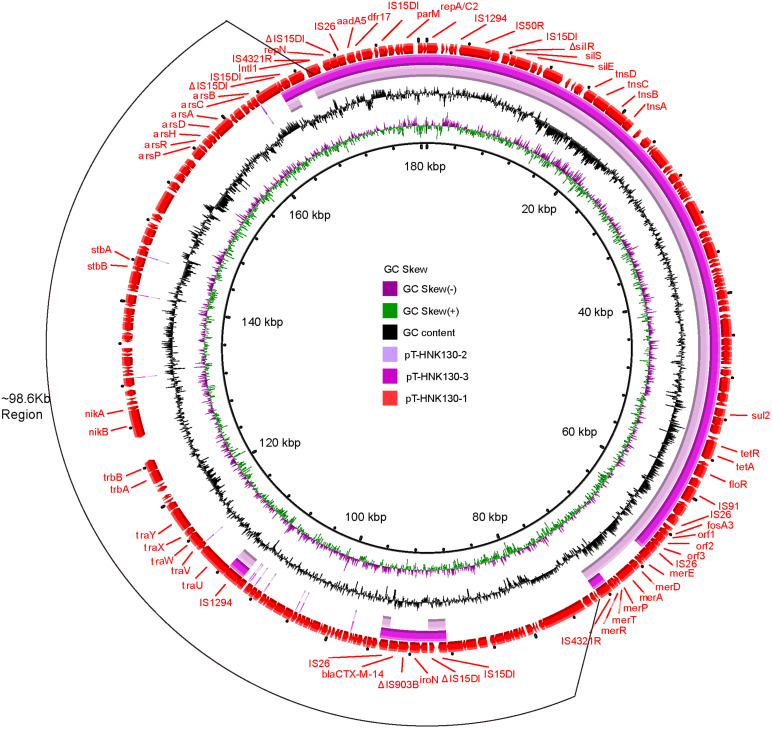
Circular alignment of plasmids pT-HNK130-1, pT-HNK130-2, and pT-HNK130-3. GC content and GC skew are indicated from the inside out. ORFs are indicated by arrows, positions and transcriptional directions of genes depicted in the outer circle belong to pT-HNK130-1, which was included as reference.

The plasmid backbone elements of pT-HNK130-1 encoded functions for plasmid replication, horizontal transfer, maintenance and stability as well as a wide range of mobile genetic elements. In addition, eight predicted ORFs were associated with resistance to aminoglycosides (*aph(6)-Id* and *aph(3*″*)-Ib*), fosfomycin (*fosA3*), phenicols (*floR*), β-lactams (*bla*_CTX–M–14_), sulfonamides (*sul1* and *sul2*), and tetracycline (*tet(A*)). These ARGs were grouped together on pT-HNK130-1 with the exception of *bla*_CTX–M–14_. This plasmid also contained six ORFs associated with resistance to mercury: *mer*A, *mer*D, *mer*E, *mer*T, *mer*P, and *mer*R (Figure 3).

**FIGURE 4:**
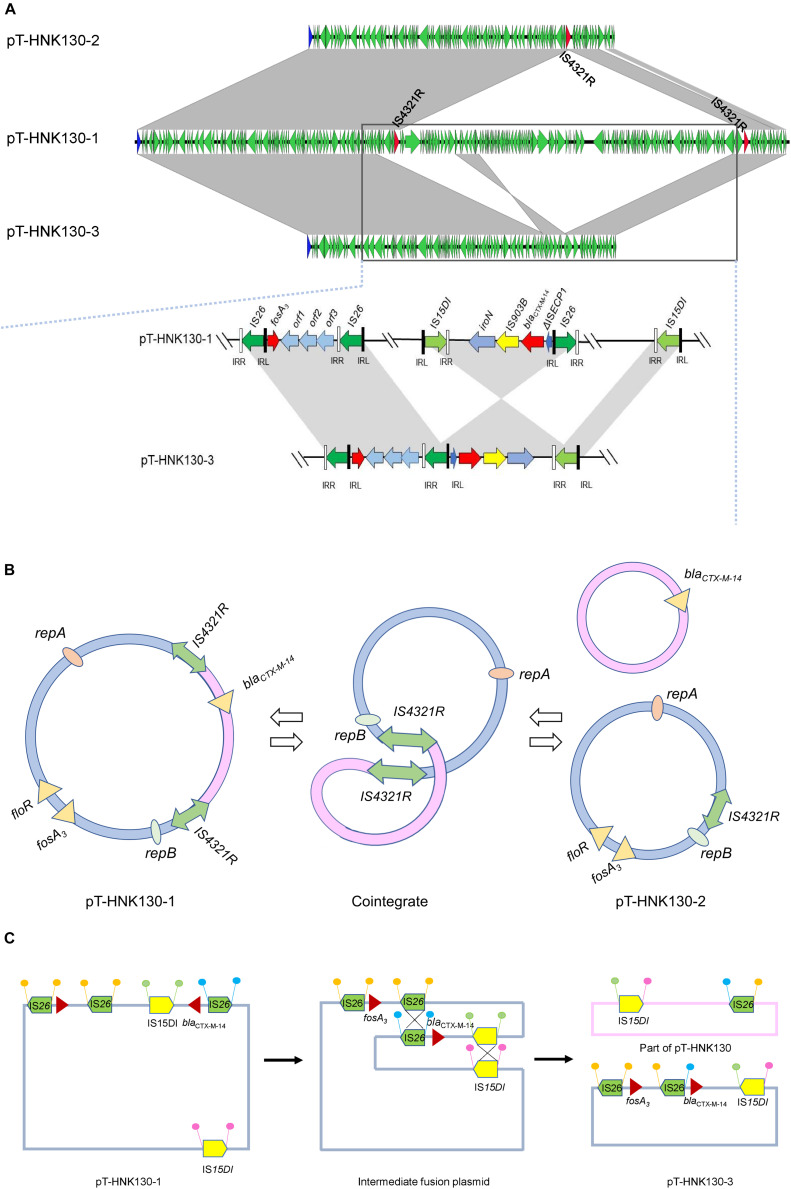
**(A)** Complete sequence linear comparison of pT-HNK130-1, pT-HNK130-2, and pT-HNK130-3; **(B)** possible mechanism of pT-HNK130-2 formation; **(C)** possible mechanism of pT-HNK130-3 formation. Boxed arrows represent the position and transcriptional direction of ORFs. Regions of >99% identity are marked by gray shading.

Genetic-environment analysis showed that the *fosA3* gene was surrounded by two *IS26* elements in the context IS*26*-322 bp-*fosA3*-1758 bp-IS*26*. In this structure, the IS*26* elements were in the same orientation forming a minicircle containing *fosA3*, and this was confirmed by inverse PCR. However, the *bla*_CTX–M–14_ gene was located on a IS*15DI-*ΔIS*15DI-iroN-*IS*903B-bla*_CTX–M–14_*-*ΔISEcp1-IS*26* structure outside the multi-resistance region (MRR) where the *fosA3* gene was located (Figures 3, 4A).

The second variant plasmid pT-HNK130-2 had a size of 85,132 bp with GC content of 52%, carried 116 ORFs, and belong to the IncC and IncN incompatibility types. Sequence analysis showed that pT-HNK130-2 was identical to pT-HNK130-1, except for a ∼98.6 kb segment between two IS*4321R*s of the pT-HNK130-1 backbone that was missing (Figures 3, 4A). The region located between two copies of *IS*4321R was 99.91% identical to pCombat13F7-3(CP019248.1) at 55% coverage. This region contained the ARG *bla*_CTX–M–14_, horizontal transfer associated genes *tra*UVWXY, and many mobile genetic elements and genes associated with plasmid maintenance. These latter were exactly the same as those missing from pT-HNK130-2 compared with plasmid pT-HNK130-1 (Figure 3).

The third variant plasmid pTHNK130-3 was 85,943 bp and composed of 119 ORFs with a GC content of 51% and belonged to the IncC and IncN incompatibility types. A linear comparison with pT-HNK130-1 revealed two segments of ∼23.7 and ∼72.7 kb that were absent from pT-HNK130-3 and *bla*_CTX–M–14_ was completely inverted (Figures 3, 4A). This led to the formation of IS*26*-*fosA3*-*orf1*-*orf2*-*orf3*-IS*26*-ΔIS*Ecp1*-*bla*_CTX–M–14_-IS*903B*-*iroN*-ΔIS*15DI*-IS*15DI* structure (Figure 4A and Supplementary Figure S3).

Based on the aforementioned sequence analysis, we designed PCR primers to distinguish between these three variants and screened 107 randomly selected transformants. Each transformant possessed only one of the three variants and the percentage we obtained for pT-HNK130-1 to pT-HNK130-3 were 65% (69/107), 14% (15/107), and 21% (23/107), respectively. Interestingly, using these primer sets with the original wild-type strain HNK130 indicated the presence of all three plasmids (Supplementary Table S3 and Supplementary Figure S4).

### Prevalence of IncC–IncN Hybrid Plasmid in *Salmonella* Isolates

These PCR primers were then used to screen 288 *Salmonella* isolates preserved in our laboratory during 2009–2014 to detect the prevalence of IncC–IncN hybrid plasmid pT-HNK130 among *Salmonella* isolates. A total of four (1.4%) isolates were detected positive for pT-HNK130. The four isolates were all recovered from chicken fecal swabs collected from 2014, but from a different location (Supplementary Figure S2). They were all resistant to fosfomycin (MIC > 256 μg/ml) and PFGE profiles were identical to that of strain HNK130. In addition, the ST and serotypes of the four strains were also the same as HNK130 and all were ST17 and serovar Indiana (Supplementary Figure S2).

## Discussion

Previous studies have reported that *fosA3* genes coexisted with *bla*_CTX–M–14_ on IncF II, IncN, IncHI2, and untypeable plasmids (Hou et al., 2012; So-Young et al., 2012; Sato et al., 2013; Yang et al., 2014; He et al., 2017). However, in the present study, the *fosA3* gene coexisted with *bla*_CTX–M–14_ on the fused plasmids that contained both IncC and IncN replicons. The IncC plasmid is a broad-host range plasmid that can be transmitted not only in *Enterobacteriaceae* but also in other Gram-negative bacteria such as *Pseudomonas aeruginosa* and *Acinetobacter baumannii* (Zhang et al., 2014). Previous studies have identified *fosA3* co-located with *bla*_NDM–1_ and *bla*_CMY–16_ on an IncC plasmid in *Salmonella* isolated from a wild animal in Germany (Villa et al., 2015; Hadziabdic et al., 2018). To the best of knowledge, our research for the first time reported the presence of both *fosA3* and *bla*_CTX–M–14_ on IncC-IncN hybrid plasmid in *Salmonella*.

The *fosA3-*positive IncC–IncN hybrid plasmid-carrying strains in this study were all MDR ST17-type *S.* Indiana even if they were collected from different provinces, which may indicate that clonal spread of ST17 *S.* Indiana was responsible for the dissemination of *fosA3*. Little information about *S*. Indiana was available until this serotype was reported in food animals and exhibited resistance to numerous ARGs (Yang et al., 2010). After this, the separation rate of *S. enterica* serovar Indiana has increased rapidly and is becoming more common in patients, retail meats and food-producing animals (Yang et al., 2010; Bai et al., 2016). In addition, the newly identified Indiana strain also showed resistance to colistin and carbapenem, the last line of defense for treating MDR infections, indicating that MDR ST17-type *S.* Indiana has become a serious problem (Wang et al., 2017).

In the present study, we completely sequenced the three variant plasmids carried by transformants obtained from parent strain HNK130 and pT-HNK130-1 was the same as the parent plasmid. This plasmid was highly similar to conjugative IncC plasmids pSE12-01738-2 and its derivative pRH-1238 that were identified in *S. enterica* serovar Corvallis isolated from a wild bird in Germany, and carried *bla*_NDM_, *fosA3*, *bla*_CMY_, and other ARGs (Villa et al., 2015; Hadziabdic et al., 2018). Compared with them, in addition to the lack of resistance genes *bla*_NDM_ and *bla*_CMY_, pT-HNK130-1 also lacked some plasmid backbone genes necessary for horizontal transfer including *traI*, *traB*, *traC*, *traN*, and *traG* and may explain why pT-HNK130 was not conjugative.

The first variant plasmid pT-HNK130-1 harbored two MRRs, one of which consisted of seven ARGs (*sul2*, *aph(3*″*)-Ib*, *aph(6)-Id*, *tetR*, *tetA*, *floR*, and *fosA3*) and six mercury resistance gens (*merA*, *merD*, *merE*, *merT*, *merP*, and *merR*). The other one possessed a single ARG *bla*_CTX–M–14_ in the IS*15DI-*ΔIS*15DI-iroN-*IS*903B-bla*_CTX–M–14_*-*ΔISEcp1-IS*26* structure, a structure similar to the genetic environments of *bla*_CTX–M–14_ in the ST131 *E. coli* isolates from hospitals in the Kyoto and Shiga regions of Japan, in which three CTX-M-14-H30 (non-Rx) isolates had an IS*26*-ΔIS*Ecp1*-*bla*_CTX–M–14_-ΔIS*903D*-IS*26*-like structure (Matsumura et al., 2015). The *bla*_CTX–M_ genes are often captured by IS*Ecp1* forming the transposition units IS*Ecp1-bla_CTX–M_-IS903D* and IS*Ecp1-bla*_CTX–M_*-orf477* that can be horizontally transferred and disseminated between different host bacteria (D’Andrea et al., 2013). Here, we present the first report of an IS*15DI-*ΔIS*15DI-iroN-*IS*903B-bla*_CTX–M–14_*-*ΔIS*Ecp1-*IS*26* structure and our sequence analysis suggest that the *bla*_CTX–M–14_ may originated from other sources via this structure, and the lack of direct repeats may have been caused by multiple transposition processes (Partridge, 2011).

The genetic environment of *fosA3* in the largest variant pT-HNK130-1 was IS*26*-322bp-*fosA3*-1758 bp-IS*26*, which was 100% identical to *fosA3* genetic structure reported in *Salmonella* isolates from a wild bird in Germany and *E. coli* isolates from healthy individuals in Japan and *E. coli* isolates from chickens in China (So-Young et al., 2012; Ho et al., 2013; Hadziabdic et al., 2018). However, another genetic environment of *fosA3* and *bla*_CTX–M–14_ was obtained from pT-HNK130-3; IS*26*-322 bp-*fosA3*-1758 bp-IS*26*-ΔIS*Ecp1*-*bla*_CTX–M–14_-IS*903B*-*iroN*-ΔIS*15DI*-IS*15DI*. The IS*26*-associated context of *fosA3* has been reported in different plasmids from multiple *Enterobacteriaceae* and has frequently been associated with several *bla*_CTX–M_ variants (So-Young et al., 2012; Sato et al., 2013; Yang et al., 2014; Lin and Chen, 2015; He et al., 2017). The genetic environment of both *fosA3* and *bla*_CTX–M–14_ genes carried by the IncC–IncN plasmid in the current study was very similar to the structure reported from *E. coli* chicken isolates in China and human clinical isolates in Korea (Hou et al., 2012; So-Young et al., 2012; Ho et al., 2013). In addition, the finding of a circular intermediate carrying *fosA3* in this study suggests that horizontal transfer of the IS*26* composite transposon might accelerate ARG dissemination. Furthermore, our results also demonstrated that the *bla*_CTX–M–14_ and *fosA3* co-transferred plasmids were stably maintained even in the absence of antibiotic. A previous study has reported that the association with *bla*_CTX–M_ likely favored the dissemination and maintenance of *fosA3* (Hou et al., 2012). Fosfomycin is used for bacterial infections including urinary tract infections caused by extended spectrum β-lactamase–producing *Enterobacteriaceae*, thus the persistence of the IS*26* transposon-like structure co-carrying *bla*_CTX–M–14_ and *fosA3* from MDR ST17-type *S.* Indiana strains may pose a new public health threat.

pT-HNK130-1 also contained a Tn7-like transposon downstream of silver resistance genes *sil*S, *sil*E, and *sil*R. Tn7-like transposons play important roles in cross-genus transfer of the *sil* and *pco* operons among *Enterobacteriaceae* (Fang et al., 2016). There is increasing concern that metal contamination functions as a selective agent in the proliferation of antibiotic resistance (Fang et al., 2016). The coexistence of metal resistance determinants and *fosA3*, and *bla*_CTX–M–14_ may facilitate *fosA3* and *bla*_CTX–M–14_ dissemination under metal selective pressures in the absence of antibiotics.

The other two sequenced variant plasmids were altered in size compared with the parental plasmid, and linear comparisons of the three variants indicated they were identical except for partial sequence deletions in pT-HNK130-2 and pT-HNK130-3. Detailed analysis based on the three plasmids enabled us to predict the possible mechanism of pT-HNK130-2 and pT-HNK130-3 formation during electro-transformation. pT-HNK130-1 and pT-HNK130-2 shared a common IS element, IS*4321R*. IS*4321R* was present in both pT-HNK130-1 and pT-HNK130-2 and could be used as a homologous recombination target to form a cointegrate whereby pT-HNK130-2 is excised (Figures 4A,B). However, the formation of pT-HNK130-3 could be a result of the fusion of pTHNK130-1 via two recombination events (double crossover) (Figures 4A,C). The DNA fragments between two directly oriented copies of IS*15DI* and IS*26* result in the exchange of these segments (Partridge, 2011). Our results revealed that homologous recombination between two copies of IS*4321R*, IS*26*, and IS*15DI*, contributes to changes in plasmid size. Plasmid recombination through IS elements is not uncommon in Gram-negative bacteria and is associated with ARG spread including the New Delhi MBL (NDM) gene (Xie et al., 2018). In addition, plasmid DNA sequences are dynamic and a single plasmid may actually be a cluster of plasmids containing heterogeneous MRRs and is a reflection of its heterogeneity. This would benefit plasmid and host survival to antimicrobial threatening conditions (He et al., 2019). In support of this, we detected all three plasmids in the parent strain using PCR. However, only one plasmid was detected in the original strain by S1-PFGE and further dissection of plasmid identities awaits further investigation.

In conclusion, this study for the first time identified and characterized a hybrid IncC–IncN plasmid carrying *fosA3*, *bla*_CTX–M–14_ as well as heavy metal resistance genes (*sil* and *mer*) in MDR ST17-type *S.* Indiana *Salmonella* isolates from chickens in China. The presence of ARGs and metal resistance genes in a single plasmid can further enhance stability in the absence of antibiotic selective pressure in the presence of metal contamination. Fosfomycin is an alternative treatment for multidrug-resistant *Enterobacteriaceae* infections in the clinic, so it cannot be excluded that this isolate may spread to humans via direct contact or the food chain. The emergence of this plasmid from *S.* Indiana isolates will seriously limit future therapeutic options and the further spread of this plasmid among *Salmonella* would be a serious public health concern.

## Data Availability Statement

The datasets generated for this study can be found in the NCBI, pT-HNK130-1 (CP045742), pT-HNK130-2 (CP046032), pTHNK130-3 (CP047128), and PRJNA631125.

## Ethics Statement

This study protocol was approved by the South China Agriculture University Animal ethics committee. The strains of food animal origin were isolated from fecal swabs of healthy chickens and the owners of the animals gave permission for their animals to be used in this study.

## Author Contributions

L-JZ wrote the manuscript, performed the experiments, and analyzed the data. X-XG, JZ, and Y-WL performed the experiments. LY and L-XF contributed to data analysis and manuscript modification. H-XJ designed the study and supervised the project. All authors contributed to the article and approved the submitted version.

## Conflict of Interest

The authors declare that the research was conducted in the absence of any commercial or financial relationships that could be construed as a potential conflict of interest.

## Footnotes

1 http://mlst.warwick.ac.uk/mlst/dbs/Senterica

2 http://rast.nmpdr.org

3 https://www-is.biotoul.fr

4 https://cge.cbs.dtu.dk/services/

5 http://blast.ncbi.nlm.nih.gov
