# Supplementary material for: Characterization of a fosA3 Carrying IncC–IncN Plasmid From a Multidrug-Resistant ST17 Salmonella Indiana Isolate
Source: Front Microbiol. 2020 Jul 21;11:1582. doi: 10.3389/fmicb.2020.01582 (PMC7385254; doi:10.3389/fmicb.2020.01582)
Supplement: Supplementary file 1 [file Data_Sheet_1.pdf]

Supplementary materials

Table S1: Primers used for detected the circular intermediate in this study

Target segment	Primer	Sequence (5' to 3')	Reference
Circular intermediate	FosA3-RC-F	TTCGGCAGGCGTCTGTTGTG	This study
	FosA3-RC-R	TTCGACTACTTTATGCGCGAG	This study

Table S2: Primers used for detect the presence of the three plasmids used in this study

Target plasmid	Primer	Sequence (5' to 3')	Reference
pT-HNK130-1	P1	F: TCGTCAGTCCAGCGATATCCC	This study
		R: TAACCAGGAGCGCTTGAACCA	This study
pT-HNK130-2	P2	F: AAACCGTGCTATCCGAAC	This study
		R: AAGCAAAATCCCGTCTGACA	This study
pT-HNK130-3	P3	F: CCGCGAACATCATCCGTTG	This study
		R: AGGAAAGCGACTATACCCACTT	This study

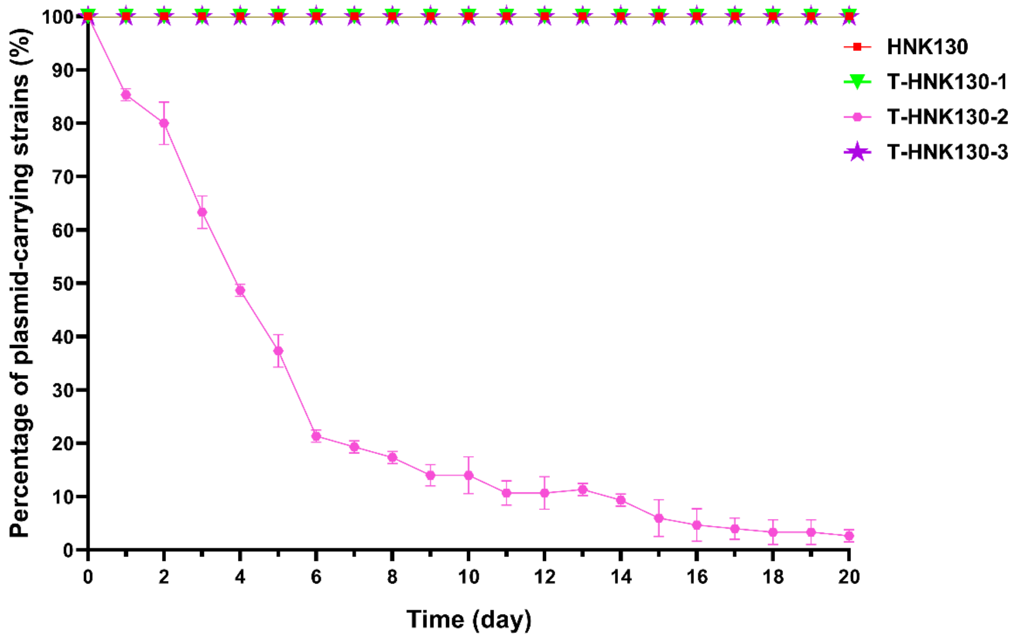

Figure S1. Measurement of plasmids stability in transformants in liquid media. Serial passaging in antibiotic-free LB broth was performed daily. All experiments were conducted in triplicate.

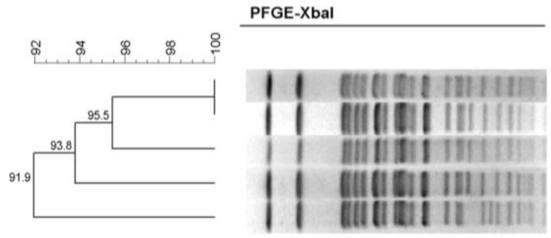

Strain	Strotype	Location[a]	PFGE type	ST type
HBA 137	Indiana	WH	A	ST17
HNJ 16	Indiana	PY	A	ST17
HNK 80	Indiana	XC	A	ST17
HBA 57	Indiana	WH	A	ST17
HNK 130	Indiana	XC	A	ST17

Figure S2. Pulsed-field gel electrophoresis finger printing patterns of Xba I-digested total DNA preparations from *Salmonella* isolates harboring *fosA3* genes. [a]Geographica locations are indicated as follows: PY, PuYang HeNan; XC, XuChang HeNan; WH, WuHan HuBei.

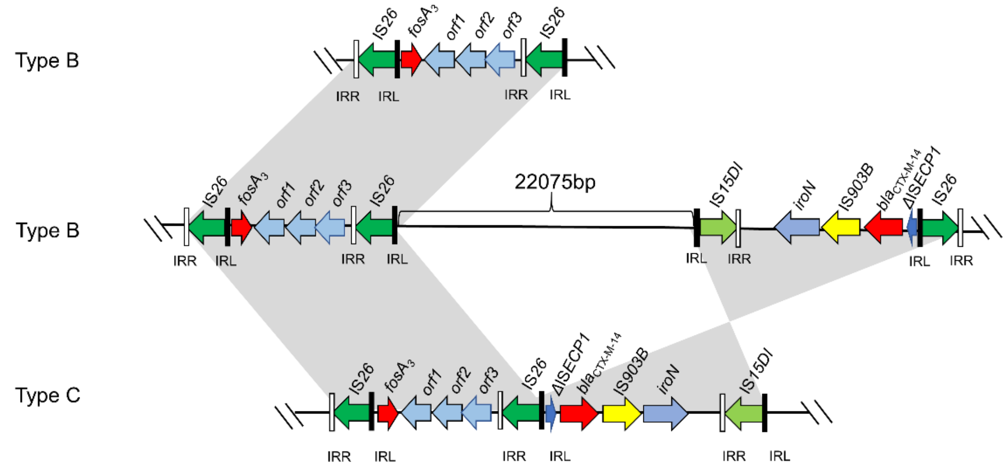

Figure S3. Three different genetic environments of *fosA3* in the plasmid in this study, regions of ≥99.0% nucleotide sequence identity are shaded gray.

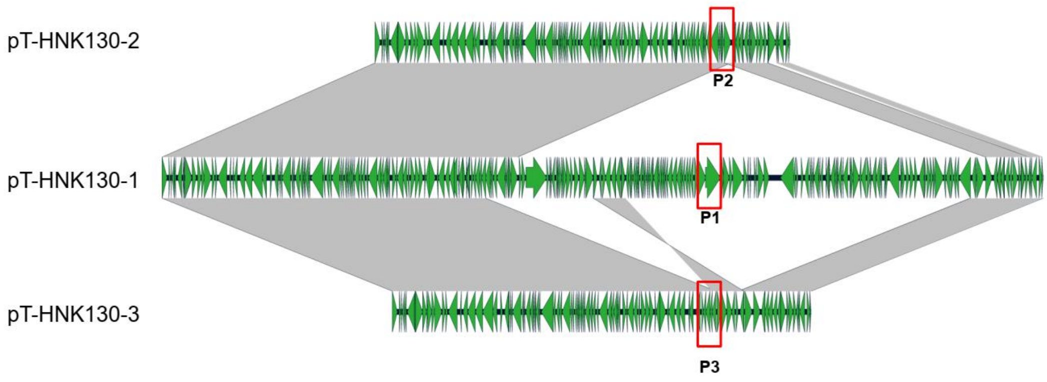

Figure S4. Amplified region for primers used to detect the presence of pT-HNK130-1, -2 and -3.
